# Supplementary material for: Characteristics, Components, and Efficacy of Telerehabilitation Approaches for People with Chronic Obstructive Pulmonary Disease: A Systematic Review and Meta-Analysis
Source: Int J Environ Res Public Health. 2022 Nov 17;19(22):15165. doi: 10.3390/ijerph192215165 (PMC9691096; doi:10.3390/ijerph192215165)

Supplementary Material

Title: "Characteristics, components, and efficacy of telerehabilitation approaches for people with Chronic Obstructive Pulmonary disease. A systematic review and meta-analysis."

S1. Table S1: Inclusion and exclusion criteria of the selected papers

Study	Inclusion Criteria	Exclusion Criteria
**Bi et al., 2021**	50 to 80 years of age regardless of gender; confirmed clinical diagnosis of stable COPD according to the standard of GOLD 2018; informed consent (patients and families); ability to use WeChat proficiently (patients and primary caregivers); ability to perform Baduanjin independently	severe heart, liver, and kidney diseases, tumors, or other conditions that may affect the observation; life expectancy less than 1 year; and history of conducting physical exercise for a long time (≥3 times/week, ≥20 minutes/time, persisting for more than 12 months)
**Bourne et al., 2017**	Diagnosis of COPD, access to the internet, and ability to operate a web platform; age ≥40y	Exacerbation requiring additional pharmacological treatment in the last 2 weeks; attendance of a pulmonary rehabilitation program within the last 6 months; respiratory disease other than COPD; uncontrolled hypertension; unstable cardiovascular disease/significant desaturation making pulmonary rehabilitation exercise unsafe or preventing program participation; inability to walk safely and independently due to non-respiratory-related conditions and/or cognitive impairment; inability to use an internet-enabled device; Timed Up and Go test >4s
**Cerdán-de-las-Heras et al., 2021**	>18 years of age; had been referred for standard rehabilitation and signed an informed consent form	Musculoskeletal abnormalities, dizziness, substantial sensory or motor impairments, dementia, and/or severe comorbidities that impeded training
**Demeyer et al., 2017**	Diagnosis of COPD; age >40; a smoking history ≥10 pack years; no current active participation in a pulmonary rehabilitation program	Presence of comorbidity limiting a normal activity pattern; the presence of another respiratory disease as the primary diagnosis; inability to understand or operate a smartphone device
**Galdiz et al., 2021**	Diagnosis of COPD, according to the GOLD guidelines with spirometric grade II-IV severity; 18 and 75 years; non-smoker and ex-smoker or smoker with an intention to quit; BODE index value between 3-7, no acute exacerbation event during the last 4 weeks	COPD with a bronchodilator response (FEV1 increase >15% of the baseline value after 200 mcg of inhaled bronchodilator), respiratory disease other than COPD, history of severe coronary artery disease, orthopedic diseases seriously limiting mobility, the life expectancy of less than 2 years, or inability to co-operate
**Hansen et al., 2019**	Diagnosis of COPD; FEV1/FVC <070, FEV1<50%; Medical Research Council ≥2	Participation in rehabilitation project within 6 months of the start of the intervention
**Holland et al., 2017**	Diagnosis of COPD; a smoking history ≥10 pack years	Presence of exacerbations within the last 4 weeks or comorbidities precluding exercise training; undertaken pulmonary rehabilitation within the last 2 years
**Jiang et al., 2020**	Diagnosis of COPD; ≥ 60 years; FEV_1/ FVC <0.7; $FEV_{1\ pred}$ <80%; use of WeChat for effective communication	Presence of mental disorders, cognitive disorders, and limb dysfunction; the presence of unstable heart disease or arrhythmia requiring drug intervention; history of myocardial infarction or cerebral infarction in the previous year; weakness influencing performance at the muscle strength test; the presence of hypertension not controlled with drugs; history of [illegible]
**Kawagoshi et al., 2015**	Diagnosis of COPD; a stable condition with no infection or exacerbation for at least the prior 3 months; ability to walk unassisted; ability to operate the device to measure their physical activity	Presence of severe and/or unstable cardiac disease, orthopedic disease, or mental disorder that could impair physical activities in daily life
**Lahham et al., 2020**	Diagnosis of mild COPD (FEV1/ FVC<70%; $FEV1_{pred}$>80%), age≥ 40y, a smoking history of at least 10 packet years; no reported hospitalizations or exacerbations in the last month	A formal diagnosis of asthma or co-morbidities that precluded exercise participation
**Langer et al., 2018**	Diagnosis of a stable condition of COPD with Pimax of < 70 cmH_20	Inability to perform physiological testing; the presence of active cardiovascular comorbidity or other conditions that could impact dyspnea or exercise capacity

**Li et al., 2022**	Diagnosis of stable PD in the first 4 weeks according to guidelines; able to complete the PR program and questionnaire survey successfully and independently; able to use WeChat proficiently	Asthma; obstructive sleep apnea syndrome; underdiagnosis of cancer; diagnosed with Alzheimer's disease or depression and anxiety disorder; having severe dysfunction of the heart, liver, or Kidney; unavailable for exercise; suffering emotional trauma in the previous 6 months such as relative death and Divorce; life expectancy less than 1 year, and history of a PR exercise for a long time (≥3 times/week, ≥20 minutes/time, persisting for more than 12
**Liu et al., 2013**	Diagnosis of a stable condition of COPD, absence of a history of bronchial asthma, a negative test for bronchiectasis, absence of oral glucocorticoid treatment in the last 3 months, and an available computer with Internet access in the home. Familiarity with logging onto the Internet, navigating to a website, and using a computer mouse; ability to watch an instruction video and graph on the computer screen and listen to instructional audio with relaxing music	Presence of malignancy, cardiac failure, distal arteriopathy, and severe endocrine, hepatic, or renal disease
**Mendes de Oliveira et al., 2010**	Diagnosis of COPD; clinical stabilityin the last 8 weeks	Hospitalization; COPD instability; the presence of neuromuscular disease, associated respiratory disease, an orthopedic or neurological disease affecting gait; recent impairment due to comorbidities
**Park et al., 2020**	Diagnosis of COPD; age ≥ 45 years; GOLD Stage range 1-3,ì; availability of a personal smartphone; using text messages; ability to communicate	Presence of psychiatric disorder; hospitalization in the last 8 weeks due to COPD exacerbation; <93% oxygen saturation in a stable state; saturation level fall to 85% after 6MWT; the presence of severe respiratory symptoms in a stable state; attendance to pulmonary rehabilitation within 12 months; the presence of other diseases that made physical activity and/or exercise difficult; using assistive devices to walk or having balance problems
**Santiworakul et al. 2021**	Diagnosis of COPD; age ≤60; 30%≤FEV_{1pred}<80%; under treatment for at least 1 year	Malnutrition; cardiovascular or neurological diseases; history of broken bones; systematic diseases; hypertension under unstable treatment or abstained from treatment for more than 6 months; condition made unstable by the rehabilitation program; more than 3 hospitalizations in the past year
**Sørensen & Svenningsen, 2018**	Diagnosis of mild to moderate COPD; home internet access via a computer, tablet, or smartphone; a PI,max equal to or less than the mean predicted PI,max for the person's gender and age group (Rochester & Arora, 1983)	Cognitive, neurological, neuromuscular, or orthopedic problems; inability to speak Danish
**Tsai et al., 2016**	Diagnosis of a stable condition of COPD; ability to operate a computer independently with adequate hearing and eyesight; weighs < 150 kg; ability to use a stationary exercise cycle indepedently; having space (at least 8 mt) in the home for a stationary lower limb cycle ergometer and walking course	Attendance of an exercise program in the last 12 months; hospitalization for an acute exacerbation of COPD in the last 2 months; no cognitive disorder; the presence of unstable cardiac or neurological disease; the presence of a home oxygen therapy; inability to understand English; living in an area without adequate internet coverage
**Vasilopoulou et al., 2017**	Diagnosis of COPD (post-bronchodilation forced expiratory volume in 1 s (FEV1)/forced vital capacity (FVC) <07) with moderate to very severe airflow obstruction (post-bronchodilator FEV1 <80% pred); age > 40 years; optimal medical treatment according to the Global Initiative for Chronic Obstructive Lung Disease; no regular use of systemic corticosteroids; a history of acute exacerbations of COPD 1 year prior to entering the study	Diagnosis of orthopedic, neurological, and other conditions significantly impairing exercise tolerance; respiratory disorders other than COPD; the presence of cognitive impairment and/or difficulties managing electronic devices that precluded interactions with the tablet
**Vorrink et al., 2016**	Diagnosis of COPD, Global Initiative for Chronic Obstructive Lung Disease (GOLD) stage 2 or 3 FEV1 30–<80%, FEV1/FVC <70% after bronchodilatation, ⩾40 years, completed pulmonary rehabilitation program of 3 months within the past 6 months; lived independently	Comorbidity greatly influencing physical activity; using an assistive device for physical activity (eg walker or mobility scooter); intermittently ceased the pulmonary rehabilitation; exacerbations resulting in hospital admission in the last 6 months
**Wang et al., 2017**	Diagnosis of COPD; FEV_1%≤80%; FEV_1/FVC ≤ 70%; clear consciousness; ability to speak Mandarin; ability to communicate; Internet and compputer available at home; able to be reached by telephone at home	Presence of comorbidities; living outside Tianjin; no access to a computer and Internet at home
**Xu et al., 2018**	Diagnosis of a clinically stable COPD; being naive to pulmonary rehabilitation	Presence of cognitive disorders, organ failure, malignant, tumors, or metabolic diseases

S2. Meta-analysis funnel plots

Figure S1. Functional capacity

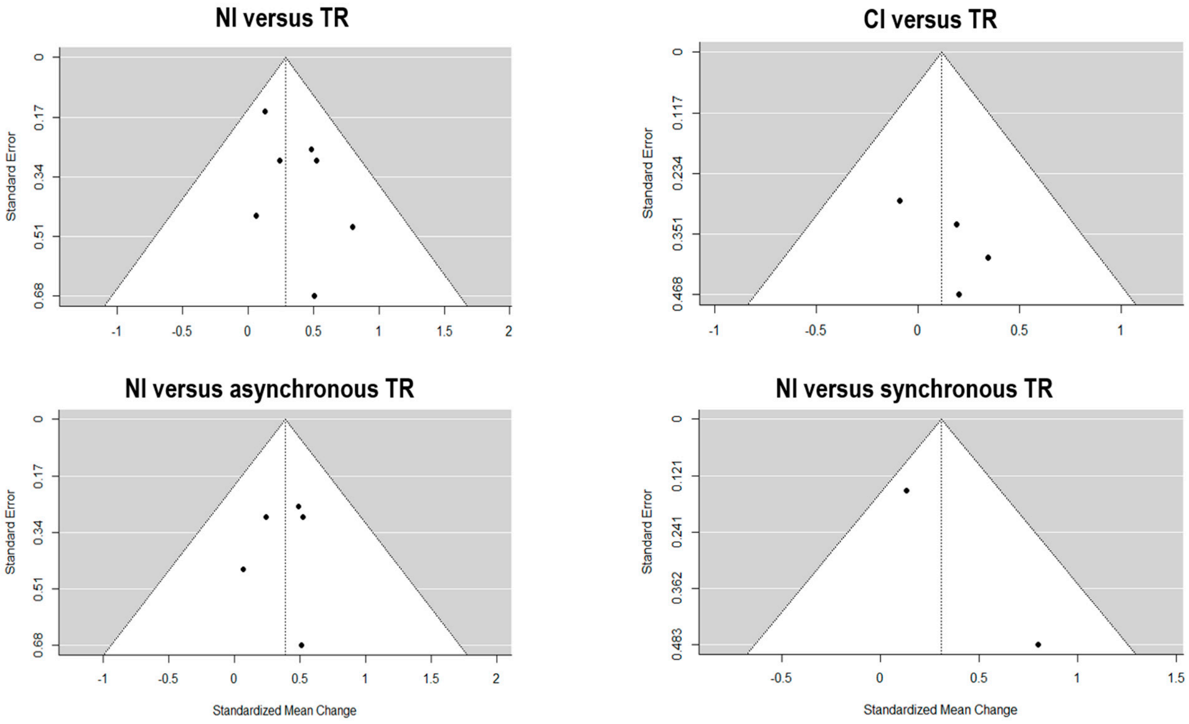

Figure S2. Dyspnoea

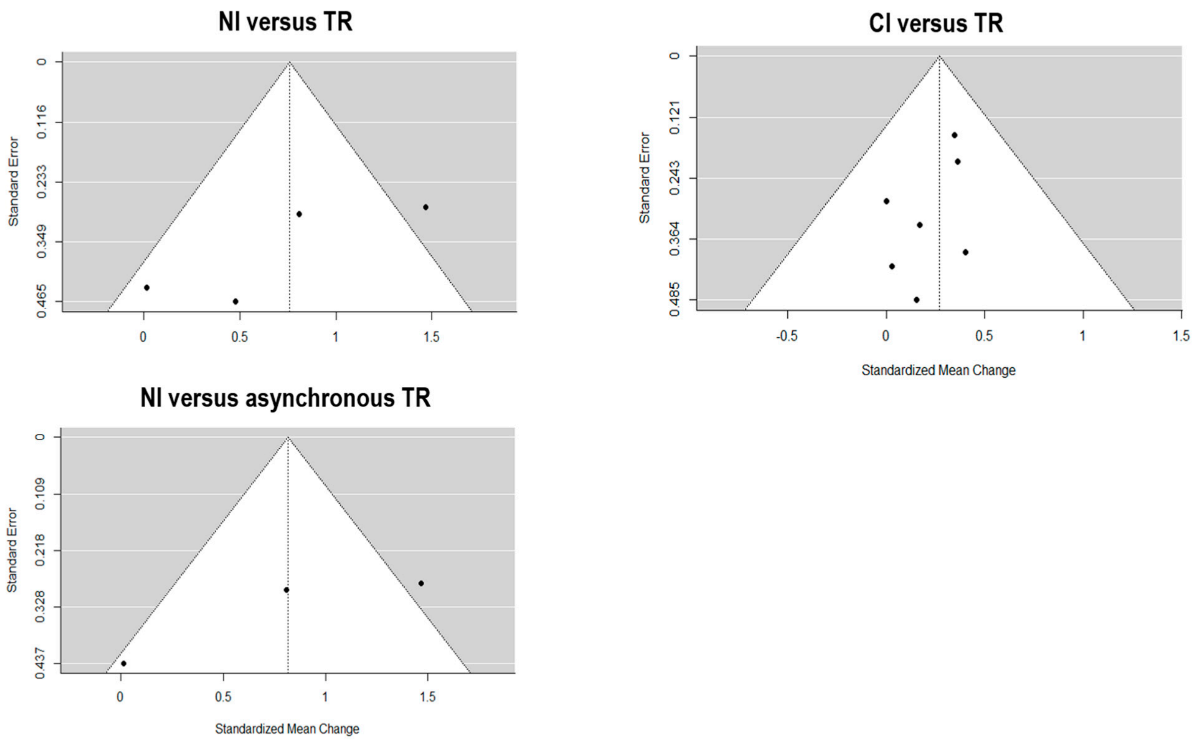

Figure S3. Quality of life

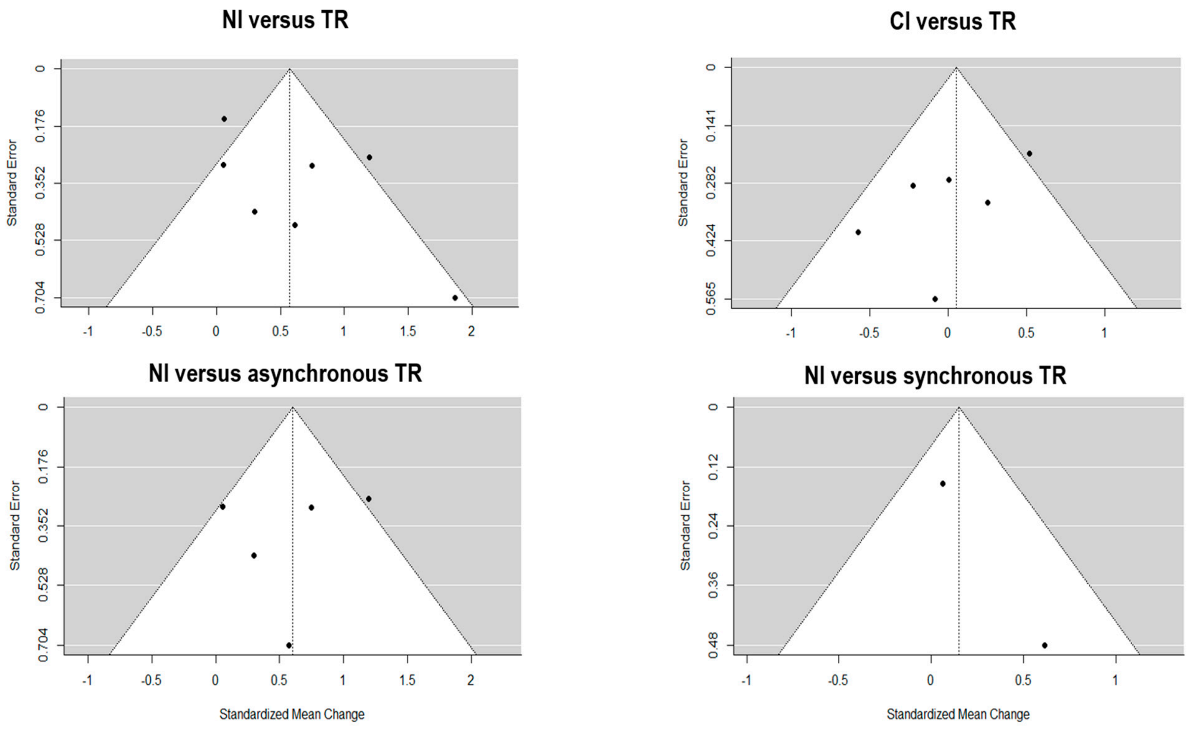

Supplement: Supplementary file 1 [file ijerph-19-15165-s001.zip › ijerph-2018951-supplementary.pdf]
